# A novel duplication frameshift mutation in the *BAG3* gene in a patient with dilated cardiomyopathy

**DOI:** 10.1186/s12872-026-05747-3

**Published:** 2026-03-18

**Authors:** Wei Zhang, Kaihang Xu, Shiguang Liu, Juan Chen, Hua Jiang

**Affiliations:** 1 Department of Cardiology, Chest Hospital, Tianjin University; Tianjin Key Laboratory of Cardiovascular Emergency and Critical Care, Tianjin Municipal Science and Technology Bureau, Tianjin, China; 2 Department of Nursing, Chest Hospital, Tianjin University; Tianjin Key Laboratory of Cardiovascular Emergency and Critical Care, Tianjin Municipal Science and Technology Bureau, Tianjin, China

**Keywords:** *BAG3* gene, Dilated cardiomyopathy, Next-generation sequencing, Sanger sequencing, Case report

## Abstract

**Background/Objectives:**

Dilated cardiomyopathy (DCM) is characterized by heart failure and cardiac dilation, distinguishing it from ischemic and non-ischemic heart diseases. To date, more than 50 genes have been identified as being associated with DCM. Mutations in the *Bcl-2-associated athanogene 3 (BAG3)* gene (NM_004281.4) play a significant role in DCM pathogenesis. However, further investigation is required for comprehensive screening of *BAG3* gene mutations. The identification of *BAG3* mutations is crucial for diagnosing DCM and elucidating its molecular mechanisms.

**Methods:**

To detect DCM-related gene mutations in the proband, next-generation sequencing was performed on DNA samples, with results subsequently confirmed through Sanger sequencing.

**Results:**

A novel duplication frameshift mutation, c.633dup (p.His212ThrfsTer43), was identified within exon 3 of the *BAG3* gene in a patient diagnosed with DCM.

**Conclusions:**

The detection of a novel duplication frameshift mutation, c.633dup, in the *BAG3* gene contributes to the expanding knowledge of *BAG3* mutations.

**Supplementary Information:**

The online version contains supplementary material available at 10.1186/s12872-026-05747-3.

## Introduction

Dilated cardiomyopathy (DCM) is characterized by left or right ventricular dilatation and systolic dysfunction in the absence of heart disease or abnormal loading conditions [1]. The condition presents with typical heart failure symptoms, including ankle swelling, progressive dyspnea, and reduced exercise capacity, and may lead to arrhythmias, sudden cardiac death, or thromboembolic events [2]. In addition to reactive pathogenesis, genetic mutations are identified in more than 35% of DCM cases [3]. While many genetic mutations exhibit autosomal dominant inheritance. X-linked, mitochondrial, or autosomal recessive inheritance patterns are less common [4]. Mutations in the *BAG3* (NM_004281.4) gene have a significant role in DCM. According to the ClinVar database (as of July 23, 2024), 1147 variants of the *BAG3* gene have been recorded. After excluding copy number increases affecting multiple genes, including *BAG3*, 1111 mutations remain, comprising 88 pathogenic, 24 likely pathogenic, and 15 classified as pathogenic/likely pathogenic mutations. However, the c.633dup mutation in the *BAG3* gene is not documented in the ClinVar database. Next-generation sequencing (NGS) utilizing target gene capture was employed to identify a novel duplication mutation (c.633dup in the *BAG3* gene), leading to a frameshift mutation extending from the 212th residue to the 255th residue, culminating in a termination codon.

## Materials and methods

### The patient

**Fig. 1:**
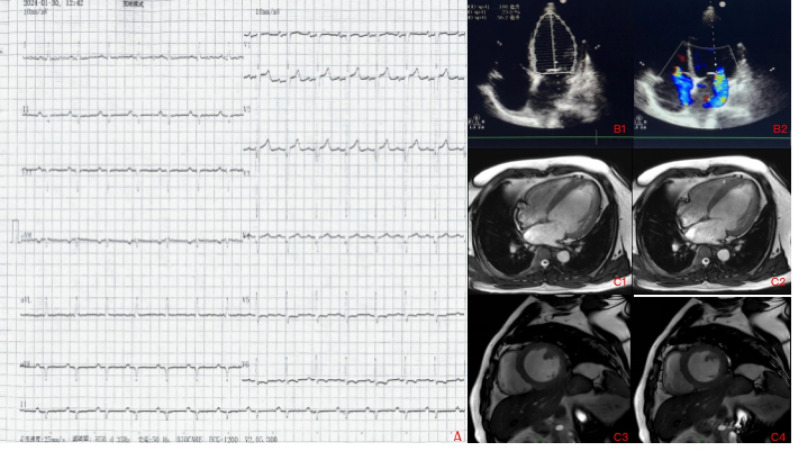
Clinical Data of the Patient. **A** Surface electrocardiogram (ECG) showing left axis deviation, sinus rhythm, reduced R-wave progression in the thoracic lead, and nonspecific ST-T changes. **B** Transthoracic echocardiography (TTE). B1: Demonstrates left ventricular dilation (left ventricular end-diastolic diameter: 70 mm) and a significantly reduced left ventricular ejection fraction (LVEF: 23%). B2: Indicates moderate mitral and tricuspid regurgitation. **C** Cardiac magnetic resonance imaging (MRI) reveals left ventricular enlargement with dyskinesia. C1–C4: Four-chamber end-diastole, four-chamber end-systole, short-axis end-diastole, and short-axis end-systole from the same imaging section. Minimal changes in left ventricular diameter between end-diastolic and end-systolic phases are observed (highlighted by the red arrow)

The proband, a 43-year-old Chinese Han male, was referred to Chest Hospital, Tianjin University, on January 30, 2024, due to worsening dyspnea over the previous 10 days. A comprehensive physical examination identified a few positive signs, including moist crackles in the lungs, cardiac boundary enlargement, and lower extremity edema. Surface electrocardiography indicated left axis deviation, sinus rhythm, reduced R-wave progression in thoracic leads, and nonspecific ST-T changes (Fig. 1A). Transthoracic echocardiography (TTE) and cardiac magnetic resonance imaging revealed left ventricular dilation and an abnormal left ventricular ejection fraction (LVEF) (Fig. 1B & C). However, computed tomography coronary angiography did not detect any abnormalities. Blood biochemistry tests were within normal limits, except for elevated levels of B-type natriuretic peptide (1918.40 pg/mL), serum creatinine (134 µmol/L), and hypersensitive troponin I (0.022 ng/mL). Prior to hospitalization, the patient had been using diuretics, β-receptor blockers, and digoxin for long-standing dyspnea. During hospitalization, dapagliflozin and vericiguat were added to the treatment regimen. Informed consent was obtained from the participant.

### Targeted sequence capture and next-generation sequencing

A gene panel comprising 312 genes was utilized for mutation detection. The DNA library was prepared using the DNA Sample Prep Reagent Set (MyGenostics, Beijing, China). Target DNA amplification was conducted in accordance with the GenCap capture kit protocol (MyGenostics, Inc., Beijing, China). Polymerase chain reaction (PCR) product purification was performed using SPRI beads (Beckman Coulter) following the manufacturer’s instructions. Sequencing of the enriched libraries was carried out using an Illumina HiSeq X Ten sequencer, employing paired-end reads of 150 bp.

The raw sequencing data were stored in FASTQ format. Cutadaptor software (http://code.google.com/p/cutadapt/) was applied to filter out low-quality reads (< 80 bp) and remove Illumina sequencing adapters. Clean reads were then mapped to the UCSC hg19 human reference genome using the BWA parameter in Sentieon software (https://www.sentieon.com/). Duplicated reads were eliminated using the driver parameter in Sentieon software, and base correction was performed to ensure that base quality scores in the BAM file closely reflected the actual mismatch probability with the reference genome. Variants were subsequently detected using the mapped reads. Single nucleotide polymorphisms (SNPs) and insertion/deletion (InDel) variants were identified with driver parameters and converted into VCF format. Variant annotation was performed using ANNOVAR software /(http://annovar.openbioinformatics.org/en/latest/), linking variants to multiple databases, including ESP6500, 1000 Genomes, EXAC, dbSNP, HGMD, and Inhouse (MyGenostics). Functional effects of variants were evaluated using PolyPhen-2, SIFT, GERP++, and MutationTaster.

### Sanger sequencing

Sanger sequencing was performed to validate the novel duplication mutation identified through target-capture next-generation sequencing (NGS). The polymerase chain reaction (PCR) primers (forward: 5’-AAGCCAGGGGAGTCATTTGT and reverse: GTCTTCTGGGCTTGGTGGAA) were designed to amplify exon 3 of the *BAG3* gene. A 26 µL reaction mixture was prepared, comprising 23 µL of Mix Golden Star T6 Super PCR Mix (1.1x), 1.5 µL of each primer, and 1 µL of DNA (about 40–50 ng), which was added to the EP tube. The genomic DNA was initially denatured for 3 min at 98 °C, followed by a thermal cycling protocol consisting of 10 s at 98 °C, 30 s at 65 °C, 10 cycles of 20 s at 72 °C, 10 s at 98 °C, 30 s at 55 °C, 30 cycles of 72 °C for 20 s, and a final elongation step at 72 °C for 5 min. The PCR products were subsequently purified and sequenced using an ABI 3730 sequencer.

## Results

**Fig. 2:**
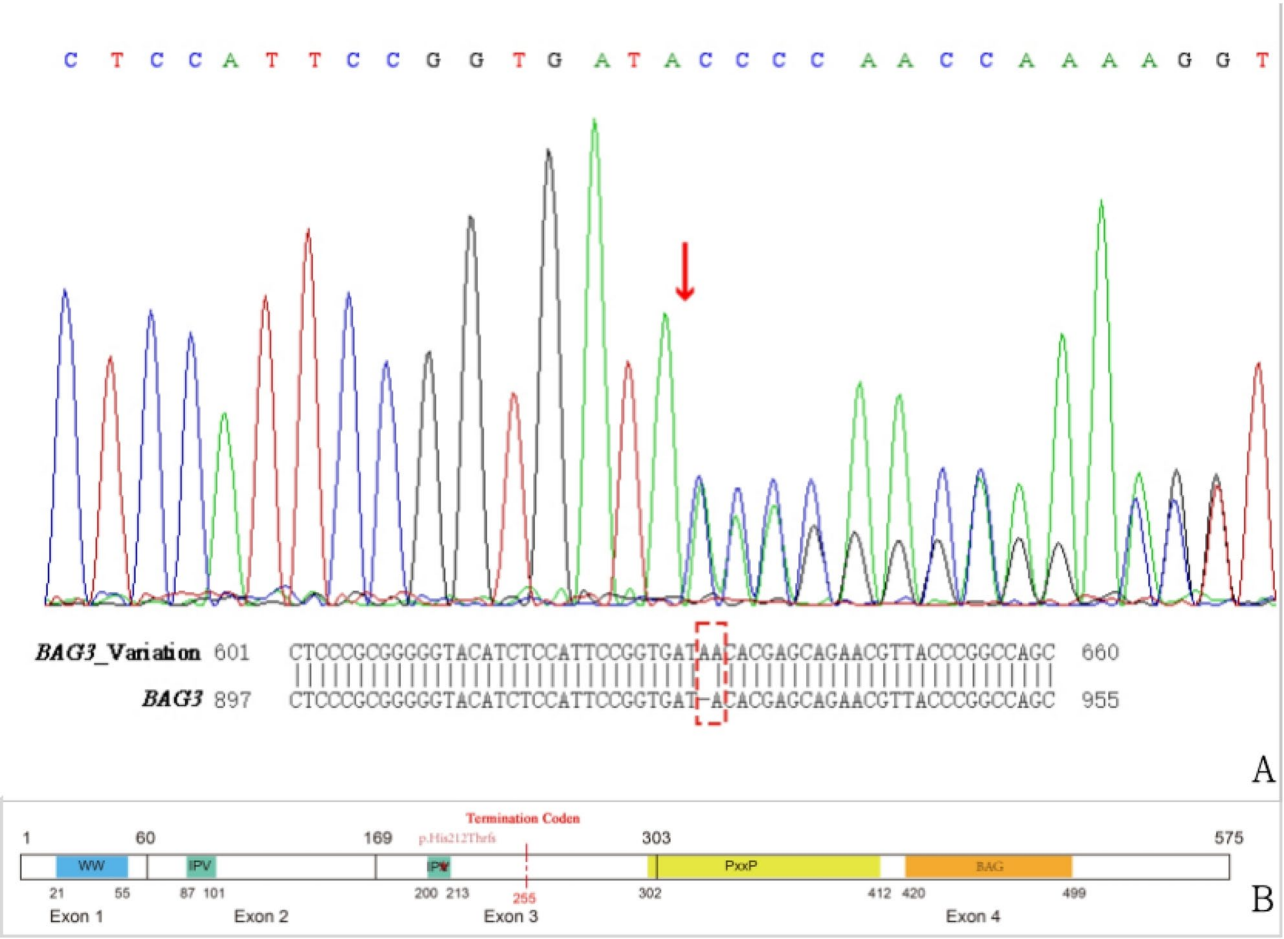
Mutation Analysis. **A** Sanger sequencing confirms a heterozygous duplication mutation, c.633dup, within exon 3 of the *BAG3* gene. The red arrow indicates the duplication site. **B** Structural representation of the *BAG3* gene, showing WW domains, two Ile-Pro-Val (IPV) motifs, proline-rich repeat (PxxP) domains, and BAG domains. The red dotted line marks the termination codon resulting from the c.633dup frameshift mutation

A total of 41 variations associated with inherited cardiomyopathies were identified through NGS. Variations with mutation reads below 5, allele frequencies exceeding 5%, synonymous mutations, or those present in the InNormal database (MyGenostics) were excluded. Following these criteria, six variations were identified (Table S1). Based on the correlation between the clinical phenotype and pathogenic classification, the exon 3 c.633dup mutation in the *BAG3* gene was considered a potential cause of DCM. The presence of this duplication mutation was further confirmed through Sanger sequencing (Fig. 2A). The mutation led to a frameshift mutation spanning from the 212th residue to the 255th residue, terminating at a stop codon (Fig. 2B). The *BAG3* gene sequence and its variant sequence are provided in the supplementary material.

## Discussion

According to the 2023 European Society of Cardiology Guidelines for managing cardiomyopathies, DCM is defined as left ventricular (LV) dilatation or systolic dysfunction that cannot be attributed solely to abnormal loading conditions or coronary artery disease. LV dilatation is characterized by a left ventricular end-diastolic diameter (LVEDD) exceeding 58 mm in men and 52 mm in women, as assessed through echocardiography. LV global systolic dysfunction is diagnosed when the left ventricular ejection fraction (LVEF) is below 50%^1^. The severity of DCM varies, ranging from mild to severe, and in some cases, it may progress to fatal outcomes. The condition primarily affects individuals between the ages of 20 and 50 years, with an estimated prevalence of 5–7 cases per 100,000 individuals annually [2]. Patients with DCM frequently seek medical attention due to dyspnea, particularly following exertion. Physical examination may reveal positive signs such as moist crackles in the lungs, cardiac boundary enlargement, and peripheral edema. Blood biochemistry findings typically include elevated B-type natriuretic peptide concentrations, while transthoracic echocardiography (TTE) demonstrates LV dilatation, systolic dysfunction, and reduced LVEF.

In this study, the proband exhibited characteristic features of DCM and was admitted due to dyspnea. TTE findings revealed an LVEDD of 70 mm and an LVEF of 23%, consistent with the established DCM criteria. Similar findings were observed through cardiac magnetic resonance imaging. Additionally, a novel duplication frameshift mutation, c.633dup in the *BAG3* gene, was identified in the proband through NGS and confirmed via Sanger sequencing. The diagnosis of DCM was established based on both genetic findings and clinical manifestations.

### The structure of the gene and function of the encoded protein

The *BAG3* gene (Fig. 2B), located on Chr10q26.11, encodes the Bcl-2–associated Athanogene 3 cochaperone and is ubiquitously expressed, particularly in the myocardium, central nervous system, and skeletal muscle. The BAG3 protein consists of four functional domains: WW (Trp-Trp) domain, two IPV (Ile-Pro-Val) motifs, PxxP (proline-rich repeat) region, and BAG domain [5]. Pathogenic variants in *BAG3* primarily affect the WW, BAG, and second IPV domains, while the PxxP domain remains largely unaffected [6].

The WW domain plays a role in activating the small GTPase Rap1, which enhances integrin-dependent cell adhesion and promotes cell viability [7, 8]. The IPV motifs interact with small heat shock proteins (HspB8/HspB6) and facilitate the formation of a ternary complex with Hsp70, which is crucial for protein degradation and refolding [9]. The BAG domain binds Bcl-2 to suppress mitochondria-mediated apoptosis and modulates caspase activation [10–12]. BAG3 also interacts with macroautophagy receptor protein 62, promoting selective macroautophagy and directing misfolded proteins to aggresomes for degradation [13].

BAG3 plays a critical role in maintaining sarcomere integrity, regulating autophagy, supporting mitochondrial function, and modulating apoptosis [14, 15]. Its deficiency has been associated with impaired β-adrenergic sensitivity and mitochondrial quality control in cardiomyocytes. Additionally, the C-terminal LEAD domain, which is conserved across mammals, inhibits apoptosis by cleaving functional caspases [5].

### Influences of mutational genes and altered proteins

DCM has been associated with more than 50 genes, some of which are implicated in its pathogenesis, including *BAG3*,* DES*,* FLNC*,* LMNA*,* MYH7*,* PLN*,* RBM20*,* SCNSA*,* TNNC1*,* TNNT2*,* TTN*, and *DSP* [16]. Among the rarer genetic causes of DCM, *BAG3* gene mutations account for about 2% of cases [17]. A recent comprehensive review has detailed the *BAG3* variants associated with DCM [6].

According to Martin et al.., BAG3 plays a crucial role in preserving cardiac sarcomere function by regulating sarcomere protein turnover. Furthermore, BAG3 haploinsufficiency can lead to sarcomere dysfunction, even when sarcomere morphology appears normal [18]. A retrospective study indicated that *BAG3* gene mutations in DCM are strongly associated with early onset heart failure and rapid disease progression. Several risk factors contribute to the poor prognosis of patients with *BAG3* pathogenic variants, including male sex, reduced LVEF, and increased LVEDD [19].

Arrhythmogenicity in BAG3-related DCM may not be as prominent as in other DCM subtypes, distinguishing it from additional genetic forms of the disease. Moreover, the treatment response in patients with *BAG3* mutations may be less favorable compared to other genetic variants of DCM [20].

### The mutational gene and changed protein

This study identified a novel duplication frameshift mutation, c.633dup (p.His212ThrfsTer43), within exon 3 of the *BAG3* gene in a patient diagnosed with DCM. A search of human gene mutation and SNP databases did not reveal any prior records of the p.His212ThrfsTer43 mutation. The detected frameshift mutation resulted in the production of incorrect amino acids from residues 212 to 254, followed by a stop codon at position 255, leading to premature protein truncation. This variant disrupted the second IPV motif and eliminated the PxxP and BAG domains.

Further genetic testing was not performed on the patient’s parents due to their concerns and psychological distress regarding the potential hereditary nature of DCM in their son. Additionally, the patient’s son did not undergo genetic screening. However, a recent cardiac ultrasound examination of the patient’s parents and son did not indicate any ultrasonographic features of dilated cardiomyopathy. An extended pedigree of the proband, along with the clinical phenotypes of first- and second-degree relatives, is presented in Supplementary Fig. 1.

This study was constrained by the limited population size and experimental conditions, which precluded further functional analysis or in vitro validation.

## Conclusions

In summary, through phenotype analysis and next-generation sequencing (NGS), a novel duplication frameshift mutation, c.633dup (p.His212ThrfsTer43), in the *BAG3* gene was identified as being associated with DCM. This mutation likely results in the production of incorrect amino acids from positions 212 to 254, ultimately leading to premature truncation of the protein.

## Supplementary Information

Supplementary Material 1.

Supplementary Material 2.

Supplementary Material 3.

## Data Availability

The raw sequence data reported in this paper have been deposited in the Genome Sequence Archive (Genomics, Proteomics & Bioinformatics 2021) in National Genomics Data Center (Nucleic Acids Res 2022), China National Center for Bioinformation / Beijing Institute of Genomics, Chinese Academy of Sciences (GSA-Human: HRA010694) that are publicly accessible at https://ngdc.cncb.ac.cn/gsa-human.

## Abbreviations

BAG3 Bcl: 2-associated athanogene 3
DCM: Dilated cardiomyopathy
Hsp: Heat shock protein
IPV: Ile-Pro-Val
LVEF: Left ventricular ejection fraction
LV: Left ventricle
LVEDD: Left ventricular end-diastolic diameter
LTCC: L-type voltage-gated calcium channel
NGS: Next-generation sequencing
PCR: Polymerase chain reaction
TTE: Transthoracic echocardiography
