# Supplementary material for: A novel duplication frameshift mutation in the BAG3 gene in a patient with dilated cardiomyopathy
Source: BMC Cardiovasc Disord. 2026 Mar 18;26:361. doi: 10.1186/s12872-026-05747-3 (PMC13122882; doi:10.1186/s12872-026-05747-3)
Supplement: Supplementary file 1 — Supplementary Material 1. [file 12872_2026_5747_MOESM1_ESM.docx]

>BAG3

MSAATHSPMMQVASGNGDRDPLPPGWEIKIDPQTGWPFFVDHNSRTTTWNDPRVPSEGPK

ETPSSANGPSREGSRLPPAREGHPVYPQLRPGYIPIPVLHEGAENRQVHPFHVYPQPGMQ

RFRTEAAAAAPQRSQSPLRGMPETTQPDKQCGQVAAAAAAQPPASHGPERSQSPAASDCS

SSSSSASLPSSGRSSLGSHQLPRGYISIPVIHEQNVTRPAAQPSFHQAQKTHYPAQQGEY

QTHQPVYHKIQGDDWEPRPLRAASPFRSSVQGASSREGSPARSSTPLHSPSPIRVHTVVD

RPQQPMTHRETAPVSQPENKPESKPGPVGPELPPGHIPIQVIRKEVDSKPVSQKPPPPSE

KVEVKVPPAPVPCPPPSPGPSAVPSSPKSVATEERAAPSTAPAEATPPKPGEAEAPPKHP

GVLKVEAILEKVQGLEQAVDNFEGKKTDKKYLMIEEYLTKELLALDSVDPEGRADVRQAR

RDGVRKVQTILEKLEQKAIDVPGQVQVYELQPSNLEADQPLQAIMEMGAVAADKGKKN

>BAG3_variation

MSAATHSPMMQVASGNGDRDPLPPGWEIKIDPQTGWPFFVDHNSRTTTWNDPRVPSEGPK

ETPSSANGPSREGSRLPPAREGHPVYPQLRPGYIPIPVLHEGAENRQVHPFHVYPQPGMQ

RFRTEAAAAAPQRSQSPLRGMPETTQPDKQCGQVAAAAAAQPPASHGPERSQSPAASDCS

SSSSSASLPSSGRSSLGSHQLPRGYISIPVITRAERYPASSPALLPPSPEDALPSAAGGV

PDPPACVPQDPGG*
