# Supplementary material for: A novel duplication frameshift mutation in the BAG3 gene in a patient with dilated cardiomyopathy
Source: BMC Cardiovasc Disord. 2026 Mar 18;26:361. doi: 10.1186/s12872-026-05747-3 (PMC13122882; doi:10.1186/s12872-026-05747-3)
Supplement: Supplementary file 2 — Supplementary Material 2. [file 12872_2026_5747_MOESM2_ESM.docx]

Supplementary figure 1. The pedigree of the patient with dilated cardiomyopathy. Squares represent males and circles represent females. Solid symbol denotes the proband with dilated cardiomyopathy. Open symbols denote unaffected individuals with clinically normal transthoracic echocardiography.
